# Highly Ordered SnO_2_ Nanopillar Array as Binder-Free Anodes for Long-Life and High-Rate Li-Ion Batteries

**DOI:** 10.3390/nano11051307

**Published:** 2021-05-15

**Authors:** Liyufen Dai, Xiangli Zhong, Juan Zou, Bi Fu, Yong Su, Chuanlai Ren, Jinbin Wang, Gaokuo Zhong

**Affiliations:** 1 Shenzhen Key Laboratory of Nanobiomechanics, Shenzhen Institutes of Advanced Technology, Chinese Academy of Sciences, Shenzhen 518055, China; lyf.dai@siat.ac.cn (L.D.); zjuan@xtu.edu.cn (J.Z.); fub@sustech.edu.cn (B.F.); cl.ren@siat.ac.cn (C.R.); 2 School of Materials Science and Engineering, Xiangtan University, Xiangtan 411105, China; xlzhong@xtu.edu.cn (X.Z.); susu0324@163.com (Y.S.); jbwang@xtu.edu.cn (J.W.)

**Keywords:** lithium-ion batteries, SnO_2_, nanoarray, anode, high-rate

## Abstract

SnO_2_, a typical transition metal oxide, is a promising conversion-type electrode material with an ultrahigh theoretical specific capacity of 1494 mAh g^−1^. Nevertheless, the electrochemical performance of SnO_2_ electrode is limited by large volumetric changes (~300%) during the charge/discharge process, leading to rapid capacity decay, poor cyclic performance, and inferior rate capability. In order to overcome these bottlenecks, we develop highly ordered SnO_2_ nanopillar array as binder-free anodes for LIBs, which are realized by anodic aluminum oxide-assisted pulsed laser deposition. The as-synthesized SnO_2_ nanopillar exhibit an ultrahigh initial specific capacity of 1082 mAh g^−1^ and maintain a high specific capacity of 524/313 mAh g^−1^ after 1100/6500 cycles, outperforming SnO_2_ thin film-based anodes and other reported binder-free SnO_2_ anodes. Moreover, SnO_2_ nanopillar demonstrate excellent rate performance under high current density of 64 C (1 C = 782 mA g^−1^), delivering a specific capacity of 278 mAh g^−1^, which can be restored to 670 mAh g^−1^ after high-rate cycling. The superior electrochemical performance of SnO_2_ nanoarray can be attributed to the unique architecture of SnO_2_, where highly ordered SnO_2_ nanopillar array provided adequate room for volumetric expansion and ensured structural integrity during the lithiation/delithiation process. The current study presents an effective approach to mitigate the inferior cyclic performance of SnO_2_-based electrodes, offering a realistic prospect for its applications as next-generation energy storage devices.

## 1. Introduction

Rechargeable lithium-ion batteries (LIBs) enjoy superior energy density and high portability, realizing their widespread utilization in our commonly used electronic devices, such as mobile phones, cameras, and laptops [1]. In the last decade, the utilization of rechargeable LIBs has rapidly expanded into the field of electric vehicles (EVs) [2]. The development of next-generation EVs requires LIBs with excellent energy density and rapid charge/discharge capability under high current densities. Currently, the commercialization of LIBs is mainly based on the carbon anodes with graphitic layered structure [3], however, the limited theoretical capacity (372 mAh g^−1^) of graphite cannot meet the requirements of next-generation LIBs, driving the exploration of alternative anode materials with high Li storage capacity. Among a wide array of anode materials, transition metal oxides (TMOs) are considered promising candidates against commercial graphite due to its rich in natural resources and outstanding Li storage capacity [4,5]. In general, the Li-storage mechanism in TMOs is either intercalation/deintercalation (M_x_O_y_ + nLi^+^ + ne^−^ ↔ Li_n_M_x_O_y_) or conversion reaction (M_x_O_y_ + 2yLi^+^ + 2ye^−^ ↔ yLi_2_O + xM) [6]. One should note that the conversion-type TMOs render high theoretical Li storage capacity, such as Fe_3_O_4_ (926 mAh g^−1^) [7], Co_3_O_4_ (890 mAh g^−1^) [8], and SnO_2_ (1494 mAh g^−1^) [9]. However, the conversion-type TMOs experience large volumetric change during the charge/discharge process, e.g., the volume change of SnO_2_ by ~300% [10,11], resulting in rapid capacity decay, inferior cyclic performance, and poor rate capability.

After the first report on the utilization of SnO_2_ as an anode in LIBs by Idota et al. in 1997 [12], extensive research has been carried out to solve the problem of volumetric expansion [13,14,15]. Benefiting from the development of nanotechnology and nanoscience [16,17,18], a wide variety of SnO_2_ nanostructures, such as nanorods [19], nanowires [20,21], nanotubes [22], and nanofibers [23], have been employed to improve the electrochemical performance and cyclic stability of SnO_2_-based anodes. Nevertheless, these nanostructures of SnO_2_ suffer from the problems of agglomeration, redundant interfaces, and limited electron transfer [24]. Hence, a highly ordered and stable SnO_2_ nanostructure is desired to overcome these problems. Herein, we have fabricated highly arrayed SnO_2_ nanopillar by anodic aluminum oxide (AAO)-assisted pulsed laser deposition (PLD) and employed as an anode electrode in LIBs. Benefiting from the unique nanoarray structure, the SnO_2_ anode rendered a reversible specific capacity of about 830 mAh g^−1^ after 300 charge/discharge cycles and maintained a specific capacity of 313 mAh g^−1^ after 6500 charge/discharge cycles at the current density of 2 C, exhibiting superior Li storage capacity and excellent cyclic life. Note that 1 C indicates the current strength of the battery when it is fully discharged in one hour, and 1 C of SnO_2_ is 782 mA g^−1^. Furthermore, it shows the discharge capacity of 433, 414, 354, and 278 mAh g^−1^ at the current density of 8, 16, 32, and 64 C, respectively, indicates the ultrahigh rate capability of as-synthesized SnO_2_ nanopillar. These results reveal the superior electrochemical performance of highly arrayed SnO_2_ nanopillar, which can be employed as binder-free electrodes in next-generation energy storage devices.

## 2. Materials and Methods

### 2.1. SnO_2_ Nanopillar Array Deposition

In order to improve the hydrophilicity of Cu foil surface and the adhesion between the AAO template and Cu foil, a plasma cleaning system was used to clean the surface of Cu foil before the transfer of AAO template. The employed AAO templates were purchased from Topmembranes Technology Co., LTD, and their thickness and aperture are around 650 nm and 310 nm, respectively. The fabrication of SnO_2_ nanopillar array included three steps: (1) transfer of AAO template, (2) SnO_2_ deposition, and (3) removal of AAO template. AAO-covered Cu foil and bare Cu foil were placed in a PLD system (Pascal Mobile Combi-Laser MBE) to deposit SnO_2_. The optimal deposition conditions were achieved by setting the laser energy at 350 mJ, laser pulse frequency at 10 Hz, substrate temperature at 500 °C and oxygen pressure at 2.0 × 10^−6^ Torr. The removal of the AAO template was accomplished by using polyimide high-temperature adhesive tape.

### 2.2. Electrochemical Characterization

The SnO_2_ nanoarray and thin-film electrodes (0.5 cm × 0.5 cm) with a mass loading of 0.12 mg/cm^2^ and 0.2 mg/cm^2^, respectively, were used for electrochemical characterization. The CR2025-type coin-cells were assembled in an Ar-filled gloves box with water and oxygen content of < 0.2 ppm. The 1 M electrolyte was prepared by adding an appropriate amount of LiPF_6_ to the mixed solution of ethylene carbonate (EC) and dimethyl carbonate (DMC) (1:1 *v*/*v*). The involved separator material is PP (polypropylene) /PE (polyethylene) /PP from Guang-dong Canrd New Energy Technology Co., Ltd (Dongguan, China). The galvanostatic charge/discharge cycling and rate capability were carried out by using a battery testing system (2001A, LAND, Wuhan, China) in the voltage range of 0.01 to 3.0 V (vs. Li/Li^+^). Cyclic voltammetry (CV) test was carried out by using an electrochemical workstation (CS2350, Wuhan, China).

## 3. Results

**Figure 1:**
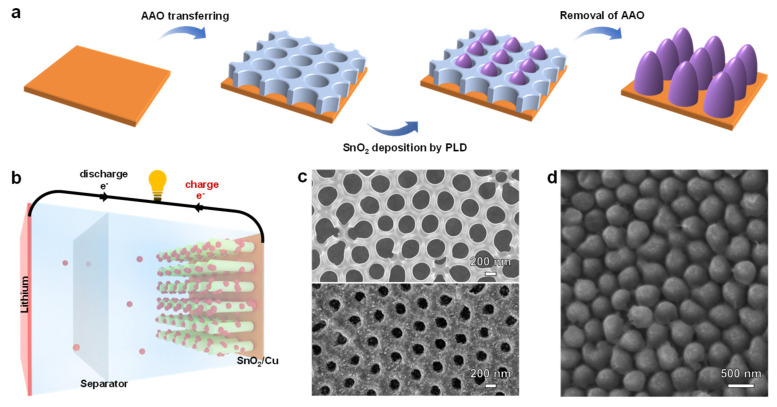
SnO_2_ array deposition. (**a**) Fabrication of SnO_2_ nanopillar array by PLD using AAO template on a Cu foil substrate; (**b**) Schematic diagram of a half-cell Li-ion battery, where SnO_2_ nanoarray is used as an anode and Li-foil is used as a cathode and counter electrode; (**c**) SEM images of bare AAO template (upper panel) and AAO template after SnO_2_ deposition (lower panel); and (**d**) a top-view SEM image of the SnO_2_ nanopillar array after the removal of AAO template.

A highly arrayed SnO_2_ nanopillar architecture has been designed to overcome large volumetric change of SnO_2_ (~300%) during the charge/discharge process. As schematically illustrated in Figure 1a, the fabrication process of SnO_2_ array includes three steps: (1) AAO template transfer, (2) SnO_2_ deposition, and (3) removal of AAO template. First, the AAO template is carefully transferred on Cu foil before SnO_2_ deposition and then, the SnO_2_ array is deposited on Cu foil by PLD using the AAO template. Finally, an array of highly ordered SnO_2_ nanopillar is left on the Cu substrate after the removal of AAO template. The utilization of SnO_2_ nanoarray as an anode material in LIBs is schematically presented in Figure 1b, where the Li-ion half-cell consists of lithium metal, the separator, and SnO_2_ nanoarray. One should note that the highly arrayed SnO_2_ nanopillar can provide enough room for volumetric change, abundant active sites for Li storage, and ordered electron transmission channels. Furthermore, the architecture of SnO_2_ array is confirmed by scanning electronic microscopy (SEM), as shown in Figure 1c,d. The top-view SEM image in Figure 1c clearly demonstrates the morphology of AAO template before and after SnO_2_ deposition, revealing the well-maintained AAO template structure during PLD. Moreover, the SEM image in Figure 1d confirms the array architecture of the SnO_2_ nanopillar, wherein the diameter of SnO_2_ nanopillar and the gap between neighboring SnO_2_ nanopillar can be estimated as ~300 nm and ~130 nm, respectively, which are consistent with the structure of AAO template. Note that SnO_2_ nanopillar with different diameter and gap were employed before we use the optimized diameter and gap as ~300 nm and ~130 nm, because we found that too small an array gap and the large nanopillar were unable to achieve long term charge/discharge cycling, which may cause by they unable to provide enough space to overcome the large volumetric change during the charge/discharge process.

**Figure 2:**
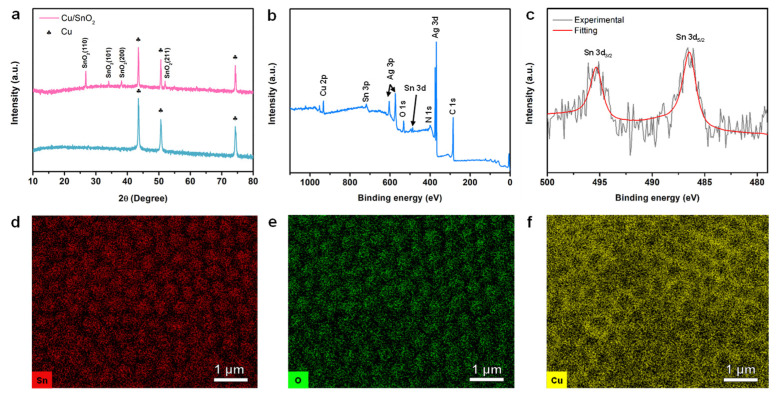
(**a**) XRD patterns of bare and SnO_2_-coated Cu foils; (**b**) wide-range XPS spectrum of SnO_2_ nanoarray; (**c**) high-resolution Sn 3d XPS spectrum; and elemental maps of (**d**) Sn, (**e**) O and (**f**) Cu from SnO_2_ nanoarray.

The X-ray diffraction (XRD) patterns of bare Cu foil and SnO_2_-coated Cu foil are presented in Figure 2a. The diffraction peaks can be well-indexed to tetragonal rutile-like SnO_2_ structure without any impurities [25]. The chemical composition of SnO_2_ nanoarray is further studied by X-ray photoelectron spectroscopy (XPS). The wide-range XPS spectrum is presented in Figure 2b, showing the presence of Sn, O and Cu elements. Note that the silver (Ag) peak appeared due to the residual silver paste, which was used during XPS sample preparation. Moreover, the high-resolution XPS spectrum of Sn 3d is plotted in Figure 2c, wherein two peaks at around 495.3 eV and 486.4 eV correspond to Sn 3*d*_3/2_ and Sn 3*d*_5/2_ [26,27], respectively, confirming the +4 valence state of Sn and formation of pure SnO_2_ phase [23]. The XPS data were further verified by energy dispersive spectroscopy (EDS). The elemental distribution of Sn, O and Cu is shown in Figure 2d-f. It can be readily observed that the Sn and O signals are prominent in the regions of nanopillar, whereas the Cu signal is relatively weaker. Overall, these results confirm the formation of a phase pure SnO_2_ nanoarray architecture.

**Figure 3:**
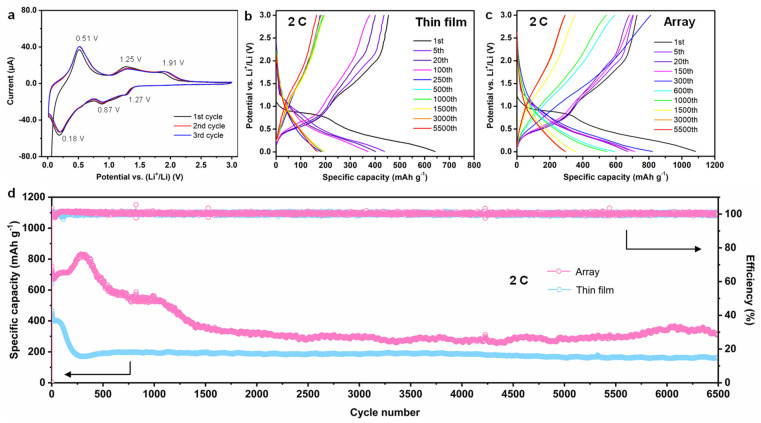
Electrochemical performance of SnO_2_ electrodes. (**a**) CV curves of SnO_2_ nanoarray at the scan rate of 0.2 mV s^−1^; charge/discharge profiles of (**b**) PLD-fabricated SnO_2_ thin film and (**c**) SnO_2_ nanoarray anodes at the current density of 2 C; and (**d**) cyclic performance of PLD-fabricated SnO_2_ thin film and SnO_2_ nanoarray at the current density of 2 C.

After structural and microstructural characterization, the cyclic voltammetry (CV) and galvanostatic charge/discharge testing were carried out to evaluate the electrochemical performance of SnO_2_ nanoarray anode in LIBs. Also, the electrochemical performance of SnO_2_ thin film is studied to demonstrate the positive influence of SnO_2_ nanoarray architecture. The first three CV curves were recorded at the scan rate of 0.2 mV s^−1^ in the voltage range of 0.01–3.0 V (vs. Li/Li^+^) to investigate the redox process in SnO_2_ nanoarray architecture. During the first cathodic scan, as shown in Figure 3a, two reduction peaks located at ~1.27 and 0.87 V (vs. Li/Li^+^) correspond to the conversion reaction of SnO_2_ to Sn (Equation (1)) and formation of solid electrolyte interphase (SEI), respectively [28,29]. Moreover, the peak at 0.18 V (vs. Li/Li^+^) is attributed to the alloying process of Sn with Li to form Li_x_Sn (Equation (2)) [30].

4Li^+^ + SnO_2_ + 4e^−^ → 2Li_2_O + Sn (1)

xLi^+^ + Sn + xe^−^ ↔ Li_x_Sn (0 ≤ x ≤ 4.4) (2)

During the first anodic process, three oxidation peaks can be observed in Figure 3a, wherein the peak at ~0.51 V (vs. Li/Li^+^) indicates the dealloying reaction of Li_x_Sn (0 ≤ n ≤ 4.4) (Equation (2)) [31], the peak at ~1.25 V (vs. Li/Li^+^) represents the decomposition of Li_x_O, and the peak at ~1.91 V (vs. Li/Li^+^) corresponds to the reoxidation of Sn [32]. The redox peaks in Figure 3a confirm the cathodic and anodic processes of the SnO_2_ nanoarray, which are consistent with previously reported SnO_2_-based anodes [20,25]. Moreover, the CV curves of SnO_2_ thin film in Figure S1 (Supporting Information) show similar cathodic and anodic peaks, confirming the successful synthesis of phase pure SnO_2_ nanoarray. As shown in Figure 3a, the overlapping CV curves after the first cycle suggest the excellent reversibility of redox reactions during the charge/discharge process [11].

Furthermore, the discharge/charge voltage profiles of SnO_2_ thin film and SnO_2_ nanoarray anode are plotted in Figure 3b,c, respectively. During the first discharge process, an apparent characteristic plateau at ~1.0 V (vs. Li/Li^+^) is found (Figure 3b), which can be attributed to the irreversible occurrence of Equation (1) [25]. Moreover, a long slope due to the formation of Li_x_Sn (Equation (2)) appeared with the further discharge process. The initial specific capacity of SnO_2_ thin film anode reached ~642 mAh g^−1^, which decreased to ~400 mAh g^−1^ and ~200 mAh g^−1^ after 100 and 250 cycles, respectively. The SnO_2_ thin film anode rendered a stable specific capacity of ~200 mAh g^−1^ from 250 to 6500 charge/discharge cycles. These results suggest that the utilization of SnO_2_ thin film as an anode in LIBs leads to excellent cyclic performance with a moderate specific capacity, which lays a solid foundation for the outstanding performance of SnO_2_ nanoarray anode. Indeed, as shown in Figure 3c, the discharge/charge plateaus of SnO_2_ nanoarray anode during the first cycle are located at the same positions as SnO_2_ thin film. Moreover, the initial discharge capacity of SnO_2_ nanoarray reached 1082 mAh g^−1^ and maintained at 700 mAh g^−1^, 600 mAh g^−1^ and 300 mAh g^−1^ after 300, 600, and 1500 charge/discharge cycles, respectively, suggesting a remarkable improvement in electrochemical performance and cyclic stability of SnO_2_ nanoarray. As presented in Figure S2, the superior electrochemical performance can be attributed to the unique architecture of SnO_2_, where highly ordered SnO_2_ nanopillar array provided adequate room for volumetric expansion and ensured structural integrity during the lithiation/delithiation process.

The excellent cyclic stability is further verified by carrying our charge/discharge cycling at the current density of 2 C (Figure 3d). In the case of SnO_2_ thin film, the reversible specific capacity was first dropped from 642 mAh g^−1^ to 402 mAh g^−1^ after 20 discharge/charge cycles and then, the specific capacity was further dropped to about 180 mAh g^−1^ during 100–250 discharge/charge cycles, which remained stable until the 6500th charge/discharge cycle. These results suggest that the SnO_2_ thin film can be used as an anode, which renders a stable but moderate specific capacity. Furthermore, the highly arrayed SnO_2_ nanopillar delivered long cycling performance and improved reversible specific capacity. As shown in Figure 3d, under the same current density of 2 C, the initial specific capacity of SnO_2_ nanoarray was found to be 1082 mAh g^−1^, which dropped to 752 mAh g^−1^ during the 2nd charge/discharge cycle.

Interestingly, the specific capacity increased to 832 mAh g^−1^ at the 300th discharge/charge cycle after a slow decay, which is even higher than the 2nd reversible capacity. A possible reason for such an anomaly is that the thick SEI layer was exfoliated due to the high surface stress during the reactivation process. Subsequently, a fresh and thin SEI layer is formed [33], which can be partly confirmed from the detailed discussion of electrochemical impedance spectroscopy (Figure S3). The newly formed thin SEI layer is more stable than the previous thick SEI layer, which can bear severe volumetric change and fracture, resulting in improved Li storage capacity and longer cycling life [34]. Note that the stable charge-transfer resistance (*R*ct) of SnO_2_ in Figure S3 reveals the well-maintained interface between SnO_2_ nanoarray and substrate. Indeed, as shown in Figure 3d, the specific capacity of SnO_2_ nanoarray gradually decreased and became stable at 524 mAh g^−1^ during the 300th to 1100th charge/discharge cycles. The attained specific capacity is two times higher than the SnO_2_ thin film. With further cycling, the reversible specific capacity stabilized at ~313 mAh g^−1^ from 2000th to 6500th cycles, demonstrating the long cycling life and high capacity of binder-free SnO_2_ nanoarray anode.

**Figure 4:**
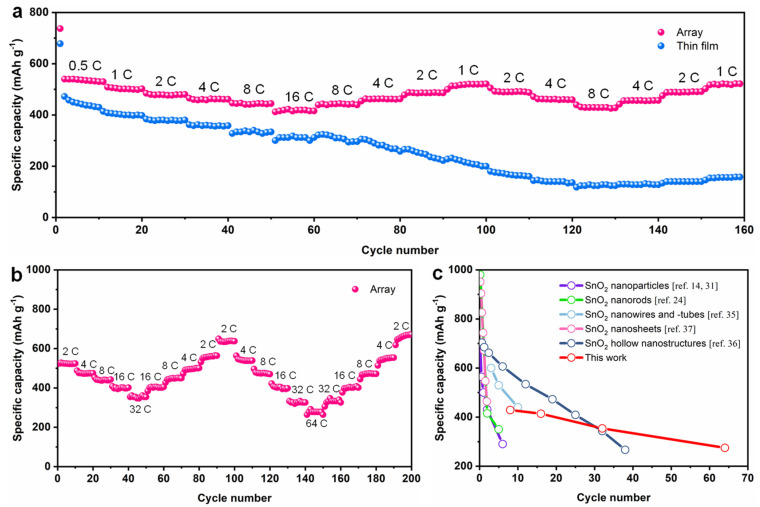
Rate performance of SnO_2_ nanoarray and SnO_2_ thin films. (**a**) The rate capability of SnO_2_ nanoarray and PLD- fabricated SnO_2_ thin film at different current densities, ranging from 0.5 C to 16 C; (**b**) rate capability of SnO_2_ nanoarray at different current densities, ranging from 2 C to 64 C; and (**c**) comparison of the electrochemical performance for SnO_2_-based anodes.

A range of current densities, i.e., 0.5 C to 16 C, was selected to investigate the cyclability of SnO_2_ nanoarray and SnO_2_ thin film anodes. Overall, the SnO_2_ thin film delivered a lower discharge capacity than SnO_2_ nanoarray, as shown in Figure 4a. In the case of SnO_2_ thin film, the discharge specific capacity decreased with increasing current density from 440 mAh g^−1^ at 0.5 C to 314 mAh g^−1^ at 16 C. The SnO_2_ thin film could not recover the initial capacity when the current density was reduced after high-rate cycling, which can be ascribed to structural failure of SnO_2_ thin film. On the other side, the SnO_2_ nanoarray anode rendered a high discharge capacity of 536 mAh g^−1^ at 0.5 C and 414 mAh g^−1^ at 16 C. More importantly, the SnO_2_ nanoarray maintained a reversible capacity of 520 mAh g^−1^ and 517 mAh g^−1^, under 1 C, after 100th and 160th cycles after high rate cycling at 16 C, indicating the excellent rate capability of nanoarray architecture. The difference in rate performance of the SnO_2_ thin film and SnO_2_ nanoarray can be attributed to the architectural differences, where the highly arrayed SnO_2_ nanopillar enjoy enough space to alleviate volumetric expansion and ensure structural stability during the charge/discharge process. 

Furthermore, we studied the rate capability of SnO_2_ nanoarray at extremely high current densities, ranging from 2 C to 64 C. As shown in Figure 4b, the SnO_2_ nanoarray delivered a specific capacity of 530, 471, 442, 398, and 354 mAh g^−1^ at the current density of 2 C, 4 C, 8 C, 16 C, and 32 C during the 1st to 50th cycle, as well as a reversible specific capacity of 556, 498, 448, 404 and 354 mAh g^−1^ at 2 C, 4 C, 8 C, 16 C and 32 C during 50th and 100th cycle, suggesting the high-rate capability of SnO_2_ nanoarray. Moreover, at an ultrahigh current density of 64 C, the SnO_2_ nanoarray delivered a specific capacity of ~278 mAh g^−1^ during the 140th and 150th cycle (Figure 4b) and then, recovered a high specific capacity of 670 mAh g^−1^ at the current density of 2 C (200th cycle). These results confirm that the SnO_2_ nanoarray anode can work under ultrahigh current densities and possess remarkable rate capability. Lastly, the electrochemical performance of binder-free SnO_2_ anode is compared with previously reported SnO_2_-based anodes [14,24,31,35,36,37], as shown in Figure 4c, demonstrating the superior Li storage capacity and high-rate capability of the as-fabricated SnO_2_ nanoarray. The superior electrochemical performance of SnO_2_ nanoarray can be attributed to the unique architecture of SnO_2_, where highly ordered SnO_2_ nanopillar array provided adequate room for volumetric expansion and ensured structural integrity during the lithiation/delithiation process. It also should note that the other two points may also play important role for our SnO_2_ nanoarray to resist the 300% volumetric change during charge/discharge process. First, we fabricate SnO_2_ nanopillar based on PLDcan ensure excellent contact between SnO_2_ and Cu, but it will also bring a strong substrate constraint from the Cu substrate to SnO_2_, which will greatly clamp the volume expansion of SnO_2_ along lateral direction, making the volumetric change less than 300%. Moreover, the obtained SnO_2_ nanopillars usually are not in uniform cylindrical shape when we use AAO-assisted PLD, as we schematic shown in Figure 1a, the top parts (away from interface of SnO_2_ and Cu) of SnO_2_ nanopillars usually prefer to appear in pyramid shape, such unique architecture provide more room and multiple routes for the expansion of SnO_2_ nanopillars.

## 4. Conclusions

In summary, we have fabricated a well-oriented and binder-free SnO_2_ nanopillar array as an anode electrode for LIBs. The as-synthesized SnO_2_ nanoarray anode rendered superior Li storage capacity of 1082 mAh g^−1^ and excellent cyclic stability (~313 mAh g^−1^ after 6500 charge/discharge cycles). Moreover, the SnO_2_ nanoarray demonstrated ultrahigh rate capability, i.e., the capacity of ~278 mAh g^−1^ at the current density of 64 C, outperforming the previously reported binder-free SnO_2_-based anodes. The superior electrochemical performance of SnO_2_ nanoarray can be attributed to the unique architecture of SnO_2_, where the highly arrayed SnO_2_ nanopillar provided adequate room for volumetric expansion and ensured structural integrity during the lithiation/delithiation process. The fabrication of SnO_2_ nanoarray presents an effective approach to enhance the energy density and rapid charge/discharge capability of LIBs.

## Data Availability

The data presented in this study are available on request from the corresponding author.

## Supplementary Materials

The following are available online at https://www.mdpi.com/article/10.3390/nano11051307/s1, Figure S1: Cyclic voltammetry curves of SnO_2_ thin films at scan rate 0.2 mV s^−1^, Figure S2: Top-view SEM images. (a,b) the top-view SEM images of as-deposited (a) SnO_2_ thin film and (b) after 5 discharge/charge cycles; (c,d) the top-view SEM images of as-deposited (c) SnO_2_ nanopillar array and (d) after 5 discharge/charge cycles, Figure S3: (a,b) the nyquist plots of SnO_2_ nanoarray (a) and thin film (b) at 20th, 50th, 400th cycles.
