# Supplementary material for: Highly Ordered SnO2 Nanopillar Array as Binder-Free Anodes for Long-Life and High-Rate Li-Ion Batteries
Source: Nanomaterials (Basel). 2021 May 15;11(5):1307. doi: 10.3390/nano11051307 (PMC8156522; doi:10.3390/nano11051307)
Supplement: Supplementary file 1 [file nanomaterials-11-01307-s001.zip › nanomaterials-1167182-supplementary.pdf]

## Supplementary Information

# Highly ordered $SnO_2$ nanopillar array as binder-free anodes for long-life and high-rate Li-ion batteries

Liyufen Dai[1,2], Xiangli Zhong[2], Juan Zou[1,2], Bi Fu[1], Yong Su[2], Chuanlai Ren[1], Jinbin Wang[2], Gaokuo Zhong[1,*]

[1] Shenzhen Key Laboratory of Nanobiomechanics, Shenzhen Institutes of Advanced Technology, Chinese Academy of Sciences, Shenzhen, China.

[2] School of Materials Science and Engineering, Xiangtan University, Xiangtan, China.

**Figure S1-S2**

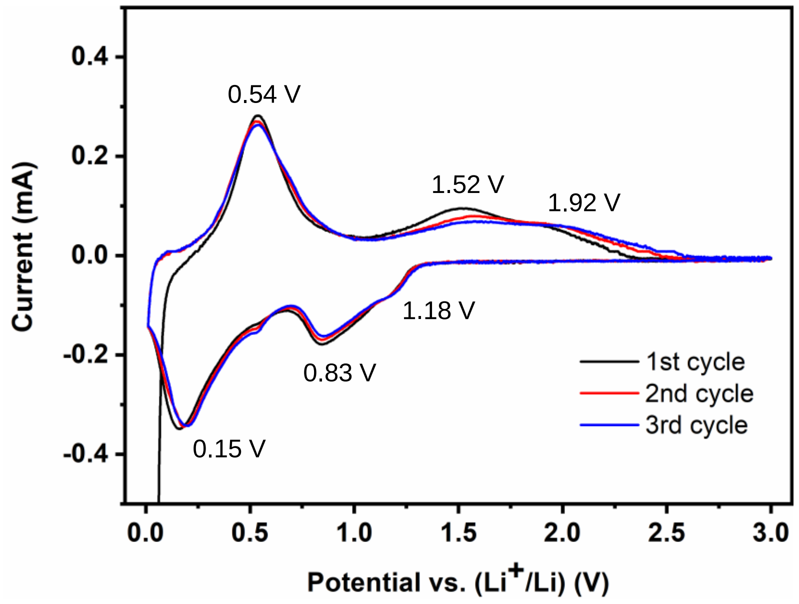


**Fig. S1** Cyclic voltammetry curves of $SnO_2$ thin films at scan rate 0.2 mV $s^{-1}$.

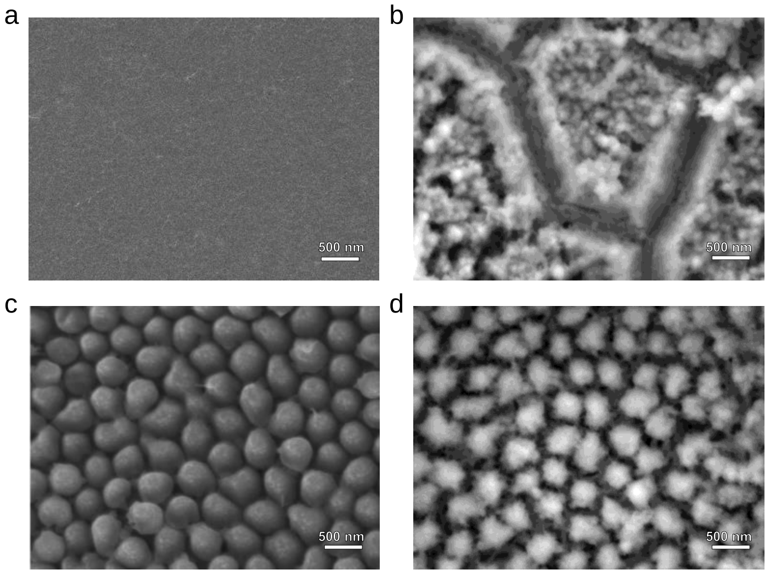


**Fig. S2 Top-view SEM images**. (a,b) the top-view SEM images of as-deposited (a) $SnO_2$ thin film and (b) after 5 discharge/charge cycles; (c,d) the top-view SEM images of as-deposited (c) $SnO_2$ nanopillar array and (d) after 5 discharge/charge cycles.

The structural changes of $SnO_2$ thin film and nanopillar array were examined before electrochemical reaction and after 5 discharge/charge cycles. As shown in Fig. S2, the surface of as-deposited $SnO_2$ thin film is smooth and uniform (Fig. S2a), while cracks appeared on the surface after 5 cycles in Fig. S2b. Regarding to the $SnO_2$ nanoarray, the architectural of highly ordered $SnO_2$ nanoarray in Fig. S2c can be well maintained after 5 discharge/charge cycles in Fig. S2d. These data support our hypothesis that well-ordered $SnO_2$ nanoarray with enough nanopillar space can effectively accommodate the volume expansion and ensure structural integrity during $Li^+$ insertion/deinsertion, and this is the reasons for our $SnO_2$ nanoarray have good energy storage capabilities.

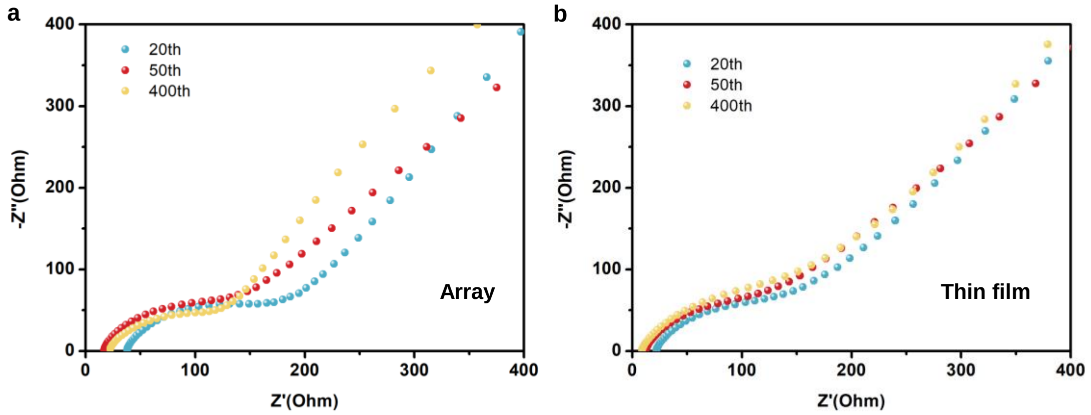


**Fig. S3** (a,b) the nyquist plots of $SnO_2$ nanoarray (a) and thin film (b) at 20th, 50th, 400th cycles.

The charge transfer and ion diffusion dynamics of $SnO_2$ nanoarray and thin films electrodes were investigated by Electrochemical Impedance Spectroscopy (EIS). Fig. S3a, b shows typical Nyquist plots, including a compressed semicircle from high to medium frequency region and a line inclined (≈ 45°) in low frequency region, wherein the compressed semicircle part represents the charge-transfer resistance ($R_{ct}$) and the line inclined part indicates ion-diffusion process (Warburg impedance ($Z_w$)). Obviously, EIS plots at 20 cycles of $SnO_2$ nanoarray displays lower $R_{ct}$ (178 Ω) than that of $SnO_2$ thin film (196 Ω), indicating $SnO_2$ nanoarray with faster process of charge transfer than $SnO_2$ thin films. The decrease of impedance suggests that the ionic conductivity of $SnO_2$ nanoarray is enhanced, benefiting for the insertion/extraction of $Li^+$. Hereafter, the $R_{ct}$ of $SnO_2$ nanoarray drops to 155 Ω after 50 cycles, which is still lower than the resistance of the $SnO_2$ thin film (186 Ω). During the subsequent cycles, the $R_{ct}$ of $SnO_2$ nanoarray declines to 149 Ω after 400th, indicating that the thicker SEI layer is exfoliated, and a more stable surface is formed. The optimized thin SEI layer is basically stable under severe volume change and fracture, contributing to rate capability and long cycle life behavior. On the other hand, the $R_{ct}$ of $SnO_2$ thin film increases to 215 Ω at the same time.
